# ATTITUDES TOWARD AUTONOMOUS VEHICLES IN RURAL OLDER ADULTS: IMPLICATIONS FOR ENHANCING ACCESS TO HEALTH CARE

**DOI:** 10.1093/geroni/igae098.0920

**Published:** 2024-12-31

**Authors:** Jong Sung Yoon, Tyler Gandy, Mojgan Zoaktafi

**Affiliations:** University of South Dakota, Vermillion, South Dakota, United States; University of South Dakota, Vermillion, South Dakota, United States; University of Illinois Urbana-Champaign, Champaign, Illinois, United States

## Abstract

Despite the potential of autonomous vehicles (AVs) to enhance health equity in rural areas, there is still a lack of understanding in rural older adults’ attitudes toward AVs. Most previous studies have identified rural older adults by relying on online survey samples, which might include individuals more likely to embrace technology and, consequently, show more favorable attitudes to AVs. Thus, the current study directly recruited a sample from rural areas (n = 120; older adults: 60+ yrs, young adults: 17 – 21yrs) to investigate two main questions: 1) whether rural older adults show negative attitudes towards AVs, and 2) whether rural older adults show positive attitudes toward AVs if they are proficient enough with technology. Based on a 16-item attitude survey which uncovered three attitudinal factors (i.e., concern with AVs, willingness to adopt AVs, confidence in AVs over human driving), we first confirmed lower willingness to adopt AVs in rural older adults compared to rural young adults (p <.01). However, in contrast to most previous studies using non-rural samples, we found neither higher concern about AVs nor lower confidence in AVs among rural older adults. Our regressions further demonstrated that higher technology proficiency predicted positive attitudes toward AVs over and above other demographic factors. The results suggest that rural older adults’ reluctance to adopt AVs may not be driven solely by concern with AVs, but rather by other reasons. We discuss the findings regarding the dissemination of AVs for enhancing access to health care for rural older adults.

